# It All Starts with a Sandwich: Identification of Sialidases with Trans-Glycosylation Activity

**DOI:** 10.1371/journal.pone.0158434

**Published:** 2016-07-01

**Authors:** Rune T. Nordvang, Christian Nyffenegger, Jesper Holck, Carsten Jers, Birgitte Zeuner, Ulrik K. Sundekilde, Anne S. Meyer, Jørn D. Mikkelsen

**Affiliations:** 1 Center for BioProcess Engineering, Department of Chemical and Biochemical Engineering, Technical University of Denmark, Kgs. Lyngby, Denmark; 2 Department of Food Science, Aarhus University, Årslev, Denmark; Russian Academy of Sciences, Institute for Biological Instrumentation, RUSSIAN FEDERATION

## Abstract

Sialidases (3.2.1.18) may exhibit trans-sialidase activity to catalyze sialylation of lactose if the active site topology is congruent with that of the *Trypanosoma cruzi* trans-sialidase (EC 2.4.1.-). The present work was undertaken to test the hypothesis that a particular aromatic sandwich structure of two amino acids proximal to the active site of the *T*. *cruzi* trans-sialidase infers trans-sialidase activity. On this basis, four enzymes with putative trans-sialidase activity were identified through an iterative alignment from 2909 native sialidases available in GenBank, which were cloned and expressed in *Escherichia coli*. Of these, one enzyme, SialH, derived from *Haemophilus parasuis* had an aromatic sandwich structure on the protein surface facing the end of the catalytic site (Phe168; Trp366), and was indeed found to exhibit trans-sialidase activity. SialH catalyzed production of the human milk oligosaccharide 3’-sialyllactose as well as the novel trans-sialylation product 3-sialyllactose using casein glycomacropeptide as sialyl donor and lactose as acceptor. The findings corroborated that Tyr119 and Trp312 in the *T*. *cruzi* trans-sialidase are part of an aromatic sandwich structure that confers trans-sialylation activity for lactose sialylation. The *in silico* identification of trans-glycosidase activity by rational active site topology alignment thus proved to be a quick tool for selecting putative trans-sialidases amongst a large group of glycosyl hydrolases. The approach moreover provided data that help understand structure-function relations of trans-sialidases.

## Introduction

The CAZy database for carbohydrate-active enzymes contains more than 280000 identified glycosyl hydrolases (GH) [1]. A recent trend in the field of glycobiology is the screening for trans-glycosylation activity among enzymes from various GH families [2]. In trans-glycosylation, the glycosyl residue retained in the covalent intermediate (CI) is attacked by a hydroxyl group of an acceptor molecule, e.g. a sugar or an alcohol, to which transfer of the glycosyl residue occurs, rather than to a water molecule as is the case in hydrolysis [3]. Thus, a trans-glycosidase can be defined as a GH whose primary activity at a given set of conditions is the transfer of a glycoside residue from a donor molecule to an acceptor molecule different from water.

In nature, saccharide synthesis is typically carried out by glycosyltransferases. However, glycosyltransferases are not suitable for industrial application *in vitro* as they require the use of nucleotide-activated glycosides as donor substrates [4]. In the trans-glycosylation reaction, however, a specific monosaccharide is transferred from one glycan (donor) to another glycan (acceptor), enabling the use of cheap biomass donors.

Despite the interest in trans-glycosidases, their rate of identification has been slow. Experimentally, GH enzymes are often identified using synthetic chromogenic substrates amenable to high-throughput screening, in which a chromophore is released irrespective of whether the monosaccharide is transferred to water (hydrolysis) or to a glycan or an aglycone moiety (trans-glycosylation). Very few screening assays for the specific identification of trans-glycosylation enzymes have been suggested [5] and consequently the vast majority of trans-glycosidases identified thus far have been identified via different routes. Therefore, it seems enticing to use bioinformatics to search for trans-glycosidases, but this strategy is hampered by the very limited number of known trans-glycosidases in different GH families. Furthermore, trans-glycosidases tend to be structurally more closely related to hydrolytic enzymes of the same GH family than to trans-glycosidases of different families, suggesting that subtle molecular adjustments rather than major modifications lead to the evolution of trans-glycosidases and questioning the existence of conserved traits among trans-glycosidases [6]. The structure-function relationship of trans-glycosidases is far from fully understood, but the importance of acceptor binding, substrate orientation, and exclusion of water from the active site has been pointed out in several cases [7–10].

As part of our quest to develop biocatalytic routes for human milk oligosaccharide synthesis, the aim of this study was to identify a trans-sialidase activity marker through the investigation of the trans-sialidase of the protozoan *Trypanosoma cruzi* (TcTS). The 3D structure and mechanism of TcTS was first reported in 2002 [9] and, due to its significant contribution to *T*. *cruzi* virulence, TcTS is to date the best characterized trans-sialidase. Other trans-sialidases have been identified, all originating from the *Trypanosoma* genus [11–14].

TcTS was identified as the ideal case study for several reasons. Firstly, it is extremely selective towards trans-sialylation with minimal hydrolytic activity [15]. Furthermore, the strictly hydrolytic sialidase of *T*. *rangeli* (TrSA), which has a sequence identity of 70% to TcTS, has recently been engineered to primarily carry out trans-sialylation under certain reaction conditions [10, 16, 17]. Trans-sialidases are also of commercial interest as it has been demonstrated that both TcTS and mutants of TrSA can efficiently sialylate a variety of glycans, enabling the production of sialylated human milk oligosaccharides [10, 16, 18].

In TcTS, two residues proximal to the active site (Y119 and W312) forming an aromatic sandwich have been reported to contribute to the trans-sialylation activity through stacking interactions with the acceptor glycan and creation of a narrow, water-excluding entry to the active site [9]. Introducing the Y119S mutation in TcTS caused an almost complete loss of trans-sialidase activity [19]. On the other hand, introduction of the opposite mutation in TrSA was not sufficient to confer trans-sialidase activity on TrSA, although the hydrolase activity was halved [19]. These results underline that Y119 is necessary, albeit not sufficient, for trans-sialidase activity in TcTS. Molecular dynamics (MD) simulations have shown the conformational flexibility of W312: when in the apo form, the catalytic cleft of TcTS is in an open form, but upon binding of a sialic acid (SA) moiety, W312 changes conformation to form a catalytic cleft, which is considerably more closed than the one of TrSA [20]. This conformational change leads to reduced solvent exposure of the TcTS covalent intermediate, which is indeed the most sensitive phase in catalysis when it comes to solvent exposure. Y119 affects W312 mobility, again emphasizing the importance of this aromatic sandwich [20]. It has also been suggested from MD simulations that conformational changes of the aromatic sandwich is responsible for expulsion of the donor aglycone leaving group from the active site followed by loading of the acceptor substrate into a position where the subsequent bond formation can take place. All this happens in a movement where W312 assisted by Y119 functions as a molecular shovel, and the aromatic sandwich thus plays a critical role in orientation of the acceptor [21]. Furthermore, Y119 has been introduced into all mutants of TrSA successfully displaying trans-sialidase activity [10, 15]. Although remarkably improved trans-sialidase activity has been obtained, the trans-sialidase activity of TcTS is still superior, underlining that the creation of a trans-sialidase–even from a well-characterized template–is not trivial [10, 15].

As an alternative, this work undertakes the task of using a rational 3D alignment approach in the search for novel, native trans-sialidases using the knowledge that the Y119-W312 aromatic sandwich is of paramount importance for trans-sialidase activity and thus a suitable trans-sialidase identification marker due to its specific structural characteristics facilitating the definition of a search motif. The hypothesis that was tested in this study is therefore: A sialidase with an aromatic sandwich proximal to the active site, as it is found in TcTS, will be a trans-sialidase. In the study an iterative *in silico* screening process a database of 2909 sialidases was gradually reduced to four candidates, one with an aromatic sandwich and three with similarly narrow binding clefts, but missing one of the aromatic sandwich residues. Recombinant expression followed by enzymatic activity studies were used to evaluate the four identified candidates. The candidate having an aromatic sandwich, a sialidase from *Haemophilus parasuis*, indeed proved to be a trans-sialidase.

## Materials & Methods

### Bioinformatics and *in silico* screening

A search motif was created to identify possible trans-sialidase candidates. Adopting the nomenclature proposed by Aasland et al. [22], the five amino acid search motif is given as ΩxRDR, where ‘Ω’ represents an aromatic amino acid and ‘x’ is any residue (Box 1). Thus, the search motif identified TcTS with W312 and R314 (bold) as shown in Box 1. The terminal residues (‘DR’) were included in the very short search motif to achieve sufficient specificity and enable a meaningful search.

**Box 1. The search motif ΩxRDR used for *in silico* screening. ‘Ω’ represents an aromatic amino acid and ‘x’ is any residue. The bottom row indicates the corresponding residues in TcTS.**

**Table:** 

Motif:	Ω x R D R
	● ● ● ●
TcTS (W312—R316):	**W** L **R** D R

The initial library dataset was assembled by acquisition of all genes in the NCBI databank matching a description of ‘sialidase’ or ‘neuraminidase’. Putative enzymes were included and redundant sequences were removed. In total, 2909 (putative) sialidases were included and screened for the presence of the motif. Candidate enzymes were modeled by the HHPred server [23] using the TcTS model (PDB ID: 1S0I) [11] as the template when possible (otherwise, the top 5 template hits were used). Subsequently, each individual model was structurally aligned to the TcTS crystal structure model using the structure alignment function of the PyMOL Molecular Graphics System (Version 1.7.4 Schrödinger, LLC).

*Micromonospora viridifaciens* neuraminidase (MvSA; PDB ID: 1W8O) [24], *Vibrio cholerae* (VcSA; PDB ID: 1W0P) [25], *Clostridium perfringens* (CpSA; PDB ID: 2VK5) [26], and *Salmonella typhimurium* (StSA; PDB ID: 3SIL) were used for analysis of the active site structure. Again, PyMOL was used for preparation of figures.

### Enzyme production

The candidate genes as they appear in GenBank were prepared for expression in the following manner: Signaling sequences were identified and removed. At the 5’ end of the protein coding sequence, a His_6_-tag sequence followed by a protease site sequence was added. The resulting genes were synthesized and inserted into the pJ414 expression vector by DNA2.0 (Menlo Park, CA, USA) allowing for IPTG-induced expression of *E*. *coli* BL21 DE3 transformed with the resulting plasmid. Transformants were picked and used to inoculate 1 L of auto-induction medium ZYM-5052 [27] containing 100 μg/mL ampicillin for 16 h with shaking at 25°C in a 5 L shake-flask. The cells were harvested by centrifugation (20 min at 5000 g) and re-suspended in binding buffer (20 mM citrate-phosphate buffer, 100 mM NaCl, 15 mM imidazole, pH 7.4). Cells were lysed by addition of 50 μg/mL lysozyme, followed by a 10 min period of incubation on ice and 6 x 30 s sonication, separated by equal breaks. The cell debris was pelleted by centrifugation at 20000 g for 20 min. The supernatant was filtered through a 0.45 μm filter and subsequently loaded onto a 1 mL Ni^2+^-sepharose gravity column and washed by addition of 10 mL of a wash buffer containing 24 mM imidazole, 100 mM NaCl and 20 mM citrate-phosphate buffer (pH 7.4). The protein was eluted from the column in 3 mL of elution buffer containing 500 mM imidazole, 100 mM NaCl and 20 mM citrate-phosphate buffer (pH 7.4). Imidazole was removed from the samples using PD10 columns (GE Healthcare, Uppsala, Sweden) following the manufacturer’s instructions. The purity of the final enzyme preparations was confirmed by SDS-PAGE.

### Time course experiment

Casein glycomacropeptide (CGMP) was obtained and purified as described previously [28]. Lactose was added to the CGMP solution to obtain final concentrations of 137.5 g/L and 50.6 g/L, respectively. Enzyme preparations were diluted to equal concentrations of total protein and an appropriate volume was heat inactivated at 90°C for 30 min. For each time point, reaction duplicates with active enzyme were started by addition of 135 μL enzyme to 365 μL substrate mix (both preheated to 30°C) and the resulting reaction mixture (100 g/L lactose, 37 g/L CGMP corresponding to approximately 4 mM 3’-bound and 4 mM 6’-bound SA) was incubated at 30°C with shaking (700 rpm). Inactivated enzymes were used as controls. Reactions were stopped by heat inactivation at 90°C for 10 min before the reaction was transferred to a 5 kDa spin filter (Sartorius AG, Göttingen, Germany) and centrifuged for 10 min at 5000 g and 4°C. As heat inactivation was unsuccessful for the SialA enzyme, these samples were stopped directly by 5 kDa filtration as described above. To evaluate the trans-sialylation activity of the enzymes, product-to-hydrolysis ratios were calculated as:

$$R_{P,H}=\frac{C_{P}}{C_{SA}}$$

where (at a given time point) C_P_ is the concentration of a given sialyllactose (SL) variant and C_SA_ is the concentration of SA. A trans-sialidase was defined as an enzyme exhibiting an R_P,H_ > 1 at any time point.

### Sample analysis by HPAEC-PAD

Concentrations of 3’SL, 6’SL and SA in the reaction permeate were measured by high-performance anion exchange chromatography with pulsed amperometric detection (HPAEC-PAD) analysis using a CarboPac^TM^ PA100 (4 mm × 250 mm) analytical column equipped with a CarboPac^TM^ PA100 (4 mm × 50 mm) guard column (Dionex Corp., Sunnyvale, CA) on a Dionex ICS-3000 system (Dionex Corp., Sunnyvale, CA). The operating conditions and analysis procedure have been described previously [17], and resulted in base line separation of the analytes. SA (Sigma-Aldrich, Steinheim, Germany), 3’SL and 6’SL (Carbosynth Ltd, Compton, UK) were used as external standards. For novel reaction products where no standard was available, the product formation was estimated using the extinction coefficient of 3’SL. As part of the identification of the novel trans-sialylation compound 3-sialyllactose, the reaction mixture was treated with β-galactosidase from *Aspergillus oryzae* (Sigma-Aldrich, Steinheim, Germany) prior to HPAEC-PAD analysis and purification by anion exchange chromatography.

### Anion exchange chromatography

Anion exchange chromatography for the purification of sialylated compounds prior to MALDI-TOF and NMR analysis was performed with an ÄKTA purifier 100 (GE Healthcare, Uppsala, Sweden) equipped with a SepharoseQ column (GE Healthcare, Uppsala, Sweden) as described previously [18].

### Matrix-assisted laser desorption/ionization—time of flight (MALDI-TOF)

A 1 μL sample and 1 μL of 10 mg/mL norharmane matrix in 50% acetonitrile was applied to a MTP 384 target plate ground steel BC (Bruker Daltonics GmbH, Bremen, Germany), mixed on the target and dried under a stream of air. The samples were analyzed with an Ultraflex MALDI-TOF/TOF instrument (Bruker Daltonics GmbH, Bremen, Germany). The instrument was operated in negative acquisition mode and controlled by the FlexControl 3.3 software package. All spectra were obtained using the linear mode with an acceleration voltage of 20 kV, and pulsed ion extraction of 10 ns. The acquisition range used was from m/z 400 to 2500. Post-source decay (PSD) spectra using the Bruker Daltonics LIFT system were recorded at 8 kV precursor ion acceleration voltage and fragment acceleration (LIFT voltage 19 kV). The reflector voltage 1 and 2 were set to 29.6 and 13.9 kV, respectively.

### NMR spectroscopy

One-dimensional ^1^H, 2D ^1^H^1^H and ^1^H^13^C NMR spectra were acquired at 298 K on a Bruker Avance III 600 spectrometer, operating at a ^1^H frequency of 600.13 MHz, equipped with a 5-mm 1H TXI probe (Bruker BioSpin, Germany). Proton and carbon chemical shifts were measured in reference to an internal acetone standard (Sigma-Aldrich; ^1^H δ 2.225 and ^13^C δ 31.08 ppm). After synthesis, samples were lyophilized and redissolved in 650 μL D_2_O (Sigma-Aldrich). 1D ^1^H NMR spectra were recorded with a spectral width of 6000 Hz, and a total of 64 scans were collected into 32 K complex data points. Water signal was irradiated using pre-saturation. ^1^H^1^H COSY spectra were recorded with 256 increments in 4096 complex data points with a spectral width of 6000 Hz. ^1^H^13^C HSQC spectra were recorded with a spectral sweep width of 6000 Hz in t_2_ and 22637 Hz in t_1_ direction. All spectra were acquired and processed using Topspin 3.0 (Bruker BioSpin, Germany). With regard to evaluation of spectra, all resonances are referenced to internal acetone (δ 2.225).

## Results & Discussion

### TcTS analysis and trans-sialidase identification strategy

**Fig 1:**
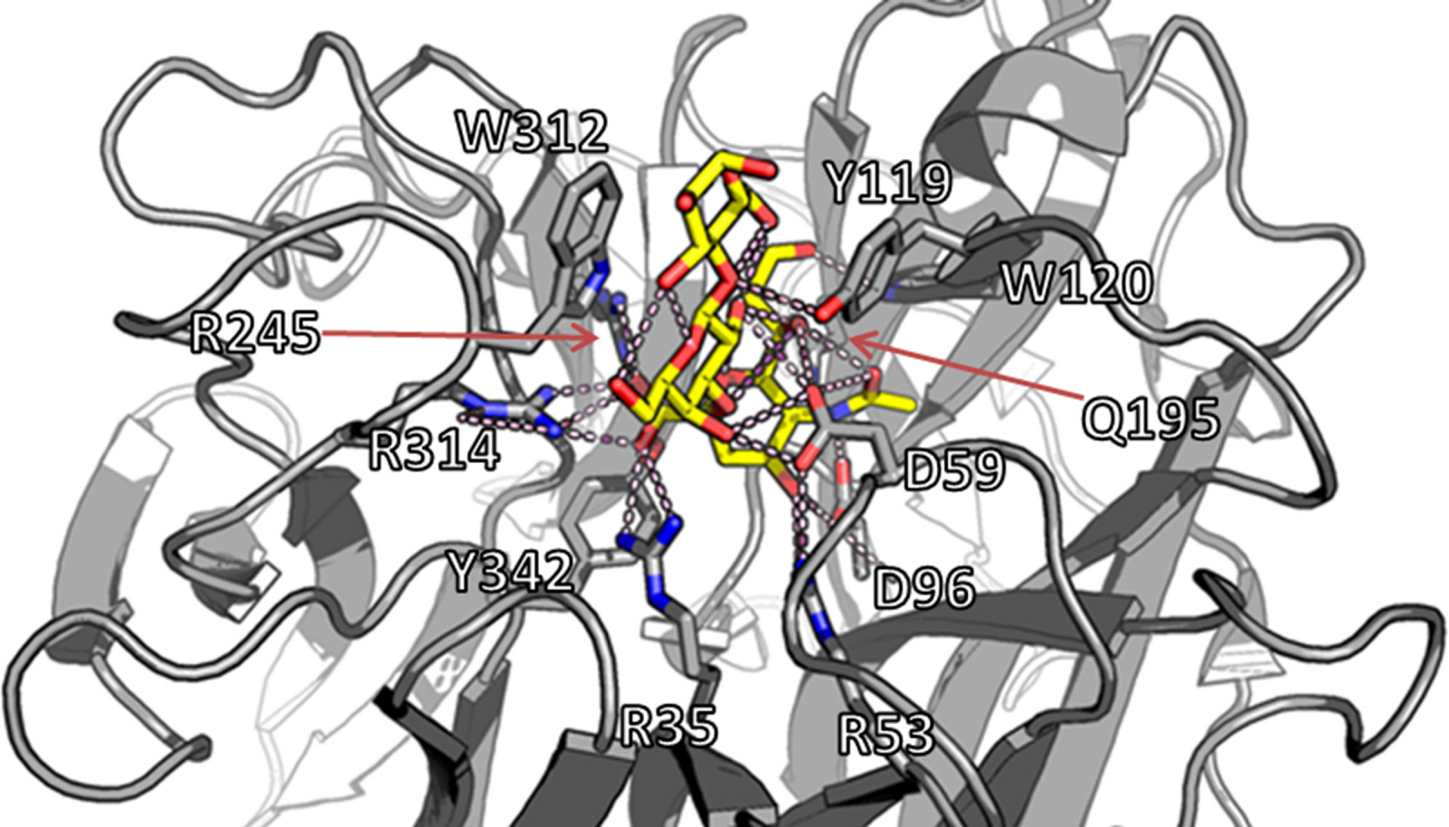
The active site of the trans-sialidase from *Trypanosoma cruzi* (TcTS). The 3’-sialyllactose substrate (yellow) is shown positioned in the active site of TcTS. The active site residues (top 9 residues in Table 1) bind the sialic acid moiety of the substrate by a number of hydrogen bonds (A), whereas the aromatic sandwich (W312 and Y119) aligns to the lactose moiety and binds it in the tight cleft above the active site (B). An overview of the interactions between enzyme and substrate is given in Table 1. The model is based on PDB entry 1S0I [11].

A visual representation of the TcTS Michaelis complex with 3’SL is presented in Fig 1 and an overview of the 11 residues interacting with the substrate in the Michaelis complex is given in Table 1 [9, 11]. Of these, R35, R245, and R314 form an Arg triad responsible for sialyl carboxylate fixation, whereas D59 is the general acid/base catalyst and Y342 acts as the nucleophile; all of these are conserved in all sialidases and trans-sialidases [9, 24]. In addition, R53, D96, W120, and Q195 also take part in sialyl fixation, while W312 and Y119 interacts with and orient the acceptor (Table 1; Fig 1). Aiming to identify new trans-sialidases, this study focused on the residues interacting in the Michaelis complex of TcTS (Fig 1; Table 1), which are conserved in all trypanosomal trans-sialidases, rather than looking for the seven amino acid residues conserved in all hydrolytic sialidases, which apart from the Arg triad, the general acid/base catalyst, and the nucleophile also include E230 acting as a base catalyst for the nucleophile and E357 stabilizing R35 through a salt bridge [9, 24]. Several factors pointed to the aromatic sandwich comprised of W312 and Y119, which are unique to trypanosomal trans-sialidases, as a good trans-sialidase identification marker. Apart from being reported to be essential for the trans-sialidase activity of TcTS, W312 in the aromatic sandwich is located close to R314, which is one of the conserved residues in all sialidases and trans-sialidases [9, 11]. The close proximity of the two residues enabled the creation of a search motif which could be used for a database search, namely ΩxRDR, where ‘Ω’ represents an aromatic amino acid and ‘x’ is any residue (Box 1).

**Table 1:** Summary of the interactions between TcTS and 3’-sialyllactose in the Michaelis complex; SA is sialyl [11, 24].

TcTS properties	Residues	Role of residues	Hydrogen bonds(to substrate)	Hydrophobic interactions	Strictly conserved
**Sialic acid moiety fixation**	R35	Carboxylate fixation	SA_O1A, SA_ O1B	-	+
	R245			-	+
	R314			-	+
	D96	Acetamide fixation	SA_N5	-	-
	W120	Glycerol fixation	SA_O9	-	-
	Q195		SA_O8, SA_O9	-	-
	R53	Ring fixation	SA_O4	-	-
**Catalysis**	D59	Acid/base catalyst	SA_O2	-	+
	Y342	Enzymatic nucleophile	(covalently bound to C2)	-	+
**Lactose moiety fixation**	Y119	Aromatic sandwich	-*	+	-
	W312		-	+	-

*Y119 goes through a conformational change during the catalytic process, where a hydrogen bond to the substrate is formed [11].

The initial database search was an identification of putative sialidases with an aromatic residue in a position relative to the conserved Arg residue where it could compose one half of an aromatic sandwich. The terminal ‘DR’ part of the motif corresponding to residues 315–316 in TcTS is not well conserved in non-trypanosomal sialidases, but the risk of losing potential trans-sialidases due to the specificity of the search motif was accepted as a premise for obtaining a manageable quantity of sequences for the subsequent homology modeling. Furthermore, false positives could be identified due to the presence of an aromatic residue not being a part of an acceptor binding site.

Therefore, a secondary screen was devised to validate the identified candidates, that is, to determine whether the other half of the sandwich was present. This was done by construction of homology models of the sequences in the reduced library. These homology models were aligned with TcTS and evaluated manually based on two criteria: 1) restoration of the active site with nine sialyl interacting residues, i.e. the seven residues involved in sialyl fixation and the two catalytic residues (Table 1), and 2) the formation of a narrow (shielded) binding cleft above the active site, preferably with an aromatic sandwich composed of two aromatic amino acid residues.

### Evaluation of initial database reduction

The initial database screening using the ΩxRDR search motif (Box 1) resulted in the identification of 46 sequences, of which 10 were redundant and 20 were sequences of already known trans-sialidases from *T*. *cruzi* and *T*. *brucei* (S1 Table). The resulting 16 was an acceptable number of sequences to enter into the next round of screening where homology models were created. Since the initial sialidase database included putative enzymes, faulty classification of enzymes identified during the sequence screening would also result in identification of false positives. However, the presence of false positives amongst the identified sequences did not pose a problem since such sequences were highly unlikely to produce homology models with an intact active site.

### Evaluation of homology models

**Fig 2:**
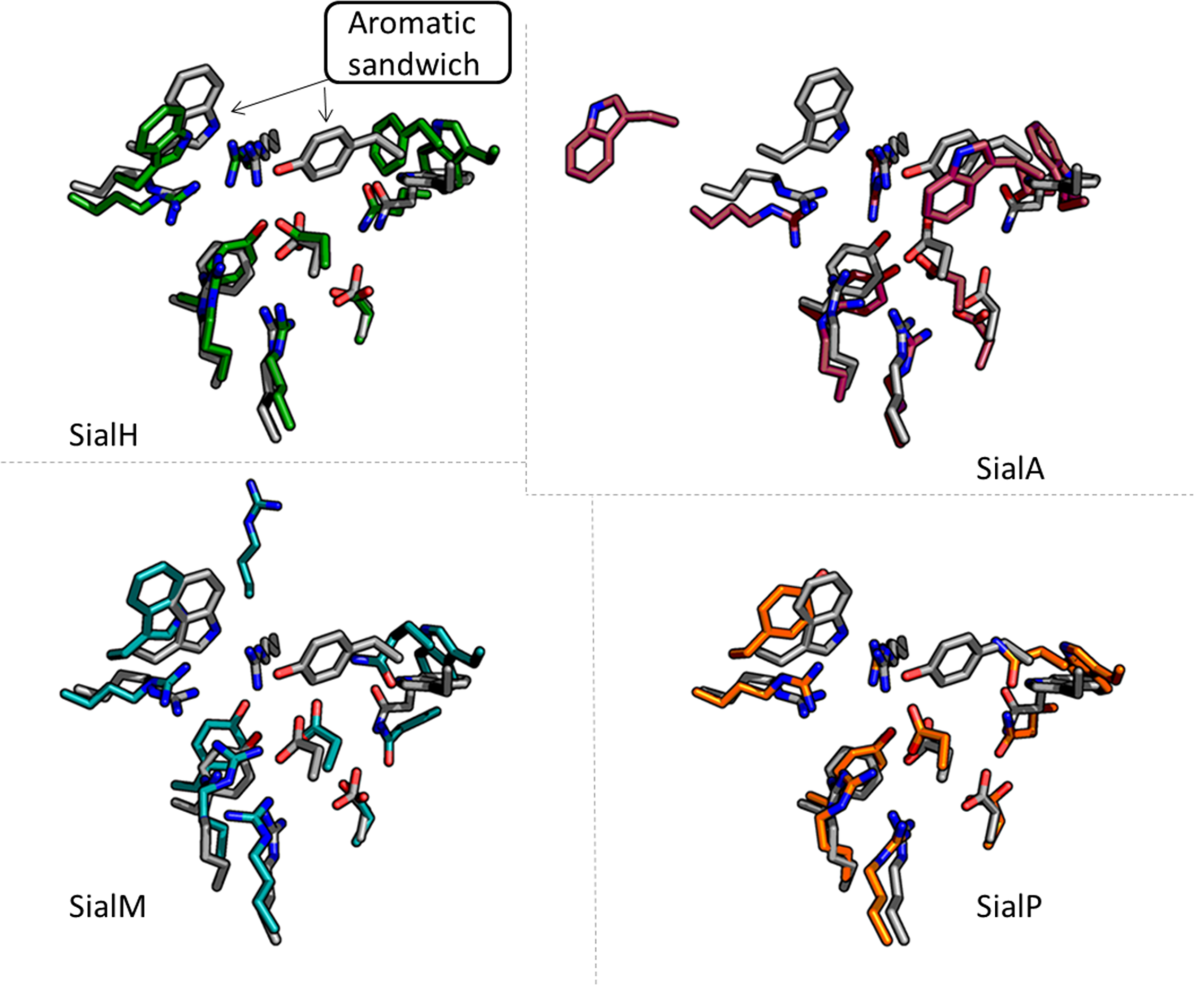
Homology modeling of active sites of the four selected trans-sialidase candidates. The four enzyme candidates selected for *in vitro* testing, SialH (green), SialA (red), SialM (blue), and SialP (orange), were modeled by homology modeling using TcTS as the template (gray). The active site and aromatic sandwich residues (indicated in TcTS with arrows in the SialH figure) are shown. The four candidates were selected based on two criteria: 1) restoration of the active site with its nine sialyl interacting residues, and 2) the formation of a narrow (closed) binding cleft above the active site, preferably with an aromatic sandwich composed of two aromatic amino acid residues. Whereas all of the selected candidates were predicted to have sterically closed active sites, SialH was the only candidate predicted to display an aromatic sandwich. The aromatic sandwich of SialH is comprised of W366 and F168 and aligns (in the model) almost perfectly to the one found in TcTS.

For two of the 16 sequences, no template for homology modeling was available (S2 Table). Homology models for the other 14 candidates were constructed and were–without regard to the quality of the models–aligned to TcTS for inspection (Fig 2; S1 Fig). Of these, 12 sequences fell for criterion 1 as they did not contain the nine sialyl interacting active site residues (S1 Fig; S2 Table). In an effort to identify any sequences that might have been missed during the initial sequence search, the two remaining sequences of the enzyme candidate models were submitted to an NCBI BLAST search and the two closest homologues were inspected to identify sequences with an aromatic residue in a position equivalent to W312 in TcTS. This led to the identification of two such sequences that were introduced in the study, modeled, and successfully evaluated against the two selection criteria (S2 Table). In total, four candidates fulfilled the two criteria, i.e. they had all nine sialyl interacting active site residues, as well as a narrow binding cleft sandwich structure, namely (putative) sialidases from *Actinomyces oris*, *Haemophilus parasuis*, *Manheimia heamolytica*, and *Pasteurella multocida*, which were named SialA, SialH, SialM, and SialP, respectively (Table 2; Fig 2). SialM, SialP, and SialH had sequence identities to each other of 58–62%, whereas SialA had sequence identities to the three others of 25–29%.

**Table 2:** An overview of the origin and selected properties of the candidate genes.

Candidate	Organism name	Uniprot ID	Submitted Name	MW	Length
SialA	Actinomyces oris/Actinomyces viscosus*	Q44562*	NanH sialidase*	73,089	685 AA
SialH	*Haemophilus parasuis*	I7AJQ9	NanH sialidase	89,948	803 AA
SialM	*Manheimia heamolytica*	E2P8L4	NanH sialidase	89,245	795 AA
SialP	*Pasteurella multocida*	I1VL53**	NanH sialidase	89,217	798 AA

*The sequence of *Actinomyces oris* (sequence ID given in S1 Table) was cloned and expressed in this work, but is not available in UniProt. Hence, the UniProt ID and submitted of the closest homologue (*A*. *viscosus*; 97% sequence identity), which was deselected out of redundancy in this work (S1 Table), is given here.

**This UniProt entry has recently been made redundant; UniProt ID Q9EZV6 lists a 51 amino acids shorter *P*. *multocida* NanH sialidase with a sequence identity of 95%.

One candidate enzyme, SialH, fulfilled both predefined selection criteria: In addition to a perfectly aligned active site, the SialH model predicted the presence of an aromatic sandwich comprised by W366 and F168, aligning to the TcTS W312 and Y119, respectively (Table 3; Fig 2). The remaining three candidates, SialP, SialA, and SialM, did not appear to harbor aromatic sandwiches. As predicted, the enzymes displayed aromatic amino acid residues corresponding to TcTS W312, but the other half of the aromatic sandwich, corresponding to TcTS Y119 was missing, although in SialA a Trp was found adjacent to the residue G404 that aligns with TcTS Y119, which could be a modeling error. Modeling errors were also suspected with respect to SialM G296 and SialA T473, since the expected amino acids were found in direct adjacency (Table 3).

**Table 3:** Summary of the 3D alignment visualized in Fig 2.

TcTS properties	TcTS	SialH	SialP	SialA	SialM
**Sialic acid moiety fixation**	R35	R80	R73	R311	R79
	R245	R298	R291	R524	*G296* (R297)^1^
	R314	R368	R359	R592	R360
	D96	D143	D136	D379	D142
	W120	W169	W162	W403	W168
	Q195	Q245	Q238	*T473* (Q472)^1^	Q243
	R53	R99	R92	R330	R98
**Catalysis**	D59	D105	D98	D340	D104
	Y342	Y402	Y393	Y620	Y394
**Lactose moiety fixation**	Y119	***F168***^3^	*N161*^2^	*G404* (W403)^1^	*Q167*^2^
	W312	W366	***Y357***^3^	W590	W358

^1^ Modeling error suspected—alternative alignment in brackets

^2^ Non-aromatic residue

^3^ Alternative aromatic residue

**Fig 3:**
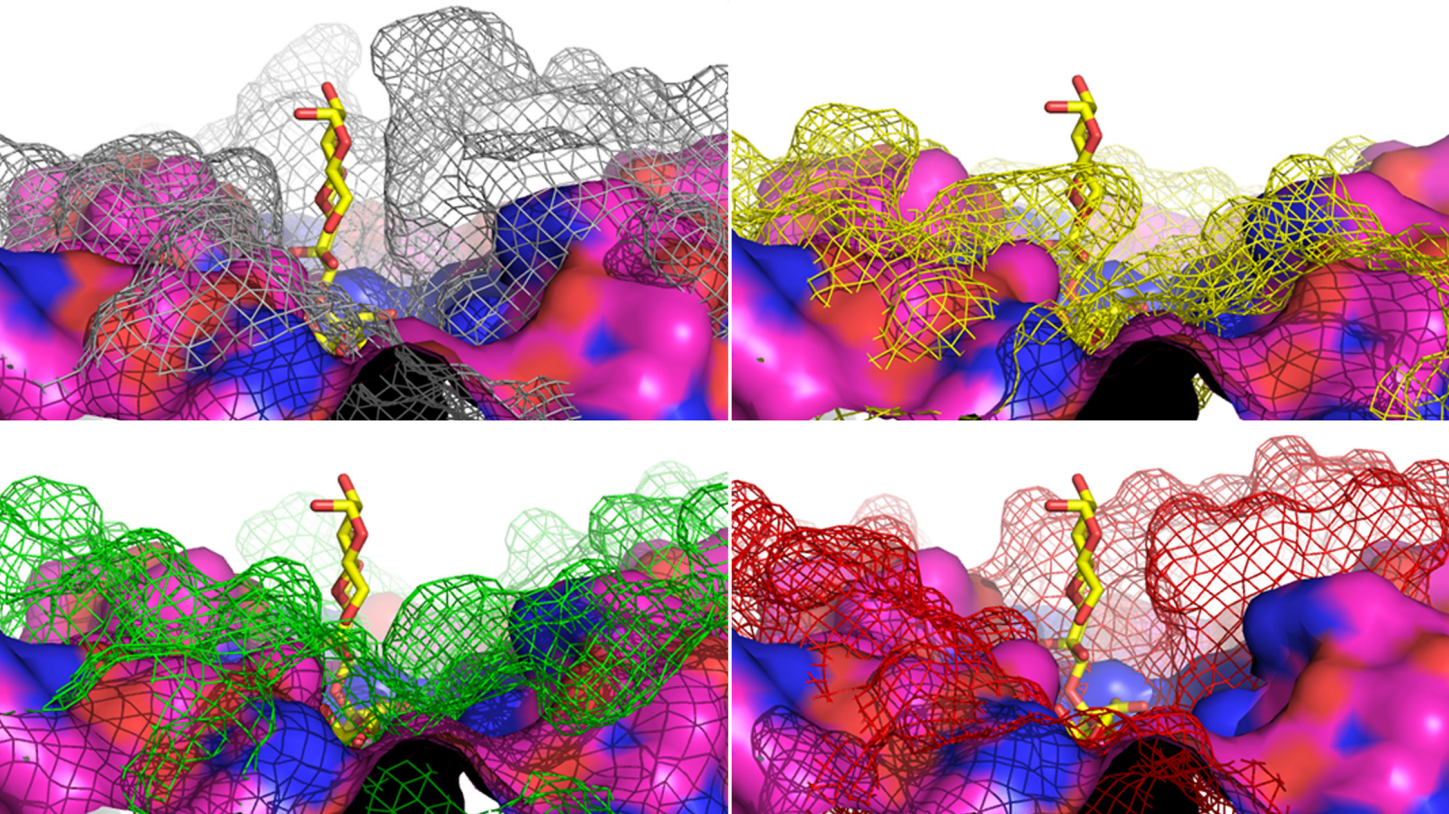
Alignment of the surface around the active site of sialidase from *Micromonospora viridifaciens* (MvSA) to TcTS and three native sialidases with limited trans-sialidase activity. The surface around the active site of sialidase from *Micromonospora viridifaciens* (MvSA; magenta surface; PDB 1W8O) with no trans-sialidase activity is aligned to TcTS (gray mesh; PDB 1S0I) in Michaelis complex with 3’-sialyllactose (3’SL; yellow sticks), and to sialidases to limited trans-sialidase activity from *Vibrio cholerae* (VcSA; yellow mesh; PDB 1W0P), *Clostridium perfringens* (CpSA; green mesh; PDB: 2VK5) and *Salmonella typhimurium* (StSA; red mesh; PDB: 3SIL), respectively.

Although an aromatic sandwich was absent in three enzymes, they were included in the study because of the closed, shielded nature of their binding clefts, which might facilitate the exclusion of water from the active site. The exclusion of water from the active site has previously been suggested to inhibit hydrolytic activity and in turn promote trans-sialidase activity in engineered sialidases, albeit by a different suggested mechanism [10]. A series of sialidase structure alignments were carried out to assert the notion that a closed active site will render trans-sialidase activity. The sialidase of *Micromonospora viridifaciens* (MvSA), which is reported to exhibit no trans-sialidase activity [29], showed a very open active site structure compared to TcTS, thus supporting selection criterion 2 (Fig 3). In addition, the sialidases of *Vibrio cholerae*, *Clostridium perfringens* and *Salmonella typhimurium* have previously been reported to exhibit limited trans-sialidase activity [30]. Indeed, their active sites are less open than the one of MvSA, but still considerably more open than the one of TcTS (Fig 3).

### Experimental characterization of trans-sialidase candidates

**Fig 4:**
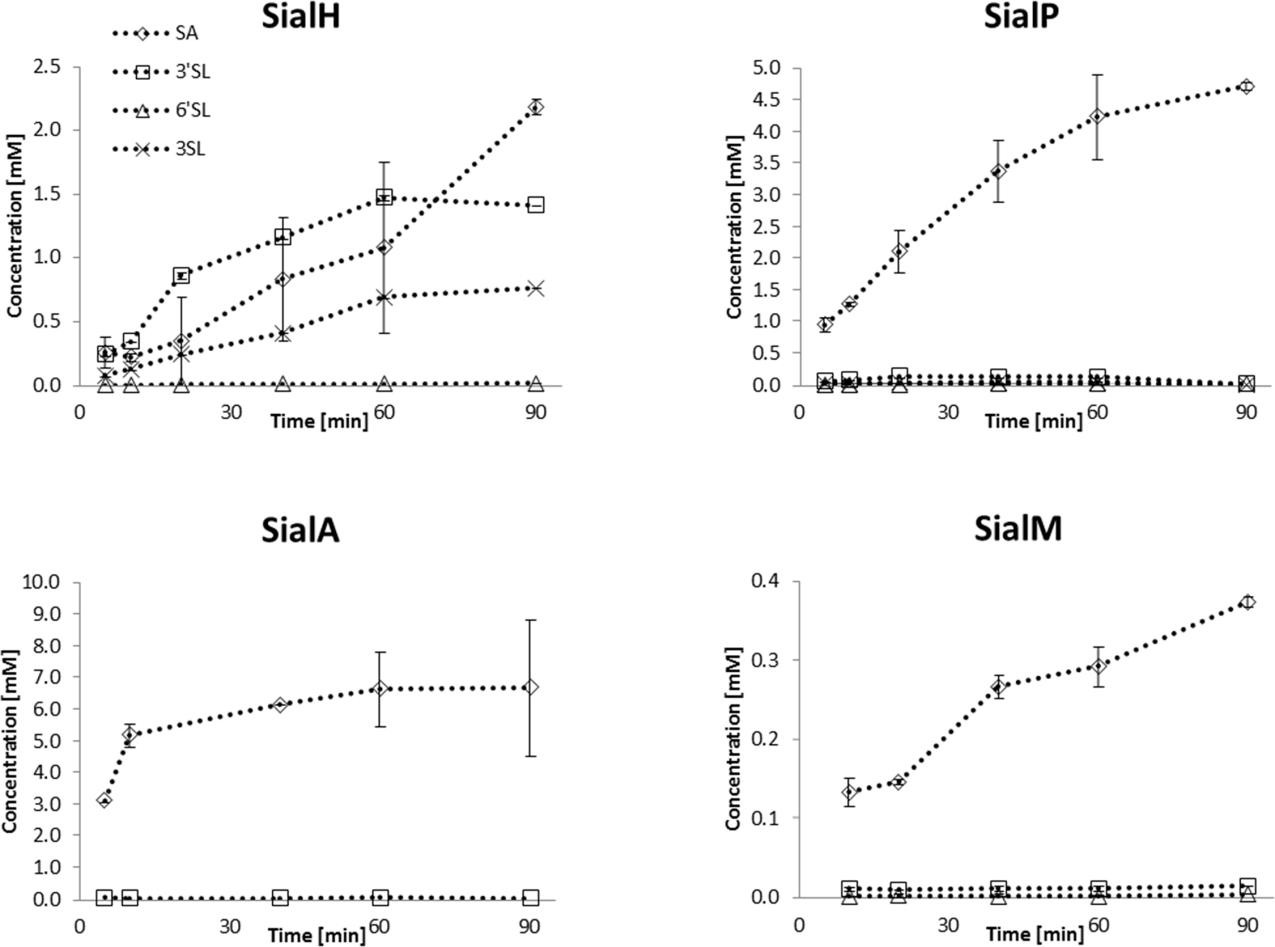
Activity of the four trans-sialidase candidates. The four candidate enzymes were used to catalyze reactions using CGMP as sialyl donor and lactose as acceptor. Formation of reaction products sialic acid (SA; all enzymes), 3’-sialyllactose (3’SL; SialH and SialP), 6’-sialyllactose (6’SL; SialP, SialA and SialM) and the novel trans-sialidase reaction product 3-sialyllactose (3SL; SialH and SialP) were monitored over a period of 90 minutes. Please note the difference in *y*-axis values. It is observed from the graphs that only SialH, which was the candidate with an aromatic sandwich, displayed a positive product-to-hydrolysis ratio. Thus, SialH can be classified as a trans-sialidase, whereas the remaining candidates are primarily hydrolytic sialidases.

**Fig 5:**
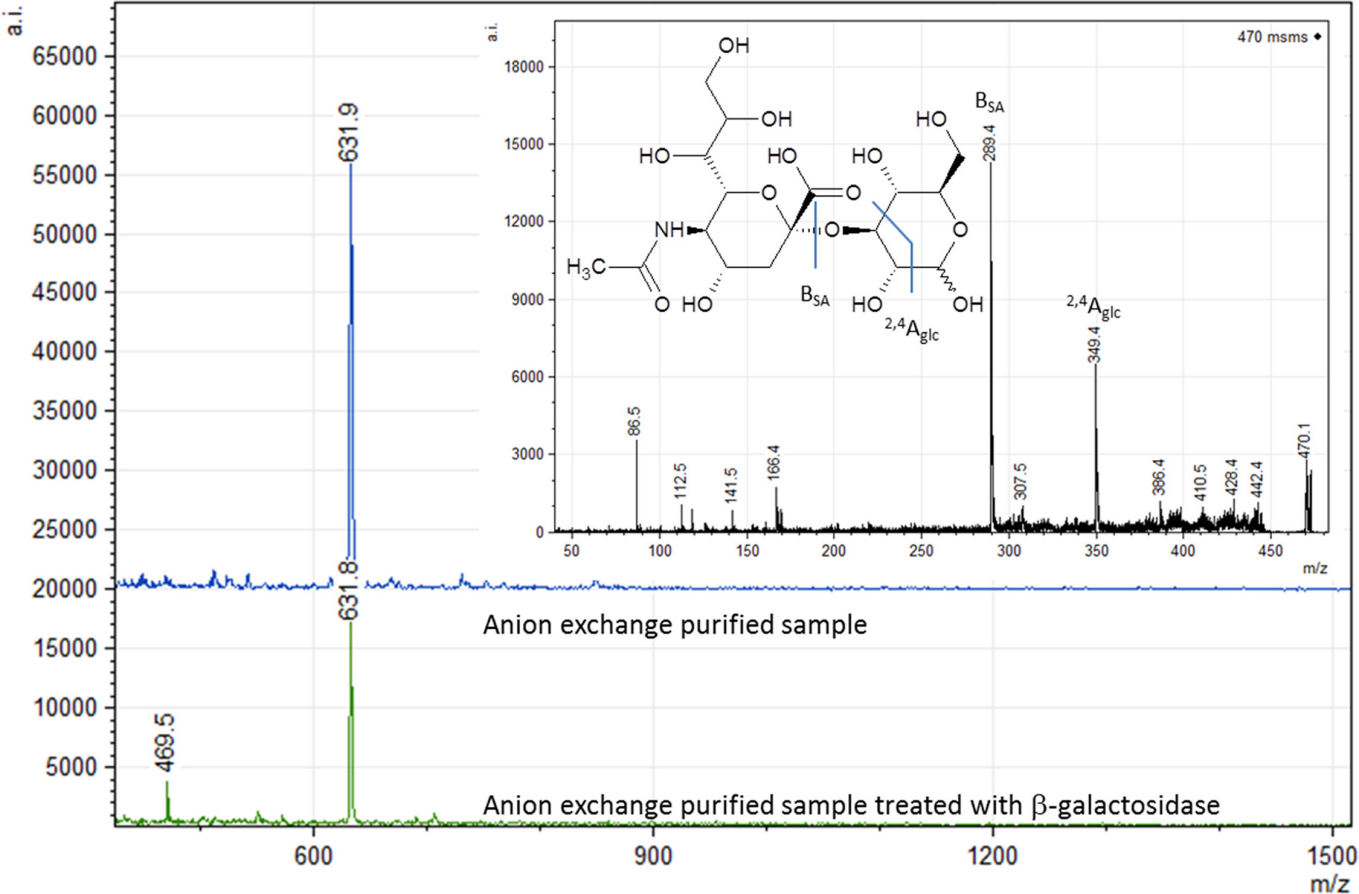
Identification of the novel trans-sialylation product by MALDI-TOF. MALDI-TOF of anion exchange purified SialH reaction mixture, i.e. all sialylated compounds in the reaction mixture, before and after β-galactosidase treatment. MALDI-TOF/TOF of m/z 632 in the untreated sample is consistent with the presence of a SL type compound. MALDI-TOF/TOF of m/z 470 ion in the β-galactosidase treated sample is the result of sialyllactose breakdown, which suggests a non-sialylated galactose moiety and thus, a sialylated glucose moiety. MS-MS of the m/z 470 ion is consistent with breakdown of sialylated glucose. Two ions resulted in significant peaks (B_SA_ = sialic acid-ion and A_glc_ = glucose-ion) and are, together with the suggested bond cleavage, indicated in the figure.

The four identified candidate enzymes were examined for trans-sialidase activity upon successful expression in *E*. *coli* as His_6_-tagged proteins. As previously, casein glycomacropeptide (CGMP) was used as donor substrate and lactose as the acceptor substrate for 3’SL production [16, 28, 31] and this reaction was performed in a time study setup, to evaluate trans-sialidase activity (Table 4; Fig 4). CGMP is a side stream product from cheese production and contains 9 mM bound α-2,3- and α-2,6-linked sialic acid in a ratio of 1:1 [32], which makes it an interesting substrate for industrial production of sialylated compounds. Our primary candidate, SialH, was capable of catalyzing trans-sialylation with a product-to-hydrolysis ratio peaking at above 2.5 for the production of 3’SL, making it the first bacterial trans-sialidase to be identified (Table 4). This novel trans-sialidase produced a relatively stable 3’SL concentration of 1.5 mM (Fig 4). Interestingly, another reaction product was formed besides 3’SL and SA. Analyzed by MALDI-TOF, the product gave rise to an m/z 632-ion suggesting that it is an SL compound (Fig 5), but HPAEC-PAD analysis ruled out that it was 6´SL (S2 Fig). The compound was susceptible to β-galactosidase hydrolysis, indicating that the compound was not sialylated on the Gal moiety at the non-reducing end (S2 Fig). The lack of sialylation at the non-reducing galactose was confirmed by NMR (S3 Fig), and the novel compound was identified as 3-sialyllactose (3SL). 3’-sialylated glycans are the primary product of trans-sialylation reactions using the *Trypanosoma* trans-sialidases, but also 6’-sialylation has been reported using other sialidases [30]. So far, 3SL is a novel trans-sialylation product.

**Table 4:** Best product-to-hydrolysis ratio (R_P,H_) for the four candidates calculated for each trans-sialylation product.

	3’SL	6’SL	3SL
**SialH**	2.5	0.010	0.70
**SialP**	0.066	0.004	0.020
**SialA**	n.a.	0.016	n.a.
**SialM**	0.088	0.020	n.a.

Product-to-hydrolysis ratios (R_P,H_) are calculated from the data shown in Fig 4; the best one obtained over the time course is shown in the Table 3‘SL: 3’-sialyllactose; 6’-SL: 6’-sialyllactose; 3SL: 3-sialyllactose.

For the three remaining enzyme candidates selected based on the narrow acceptor site, only very limited trans-sialylation activity was observed and they primarily hydrolyzed the donor-bound SA (Fig 4; Table 4). Product specificity in the trans-sialylation varied among the candidates, which is not uncommon for this class of enzymes [30]: SialP preferentially catalyzed synthesis of 3’SL, but the novel product 3SL was also observed. SialM preferred synthesis of 3’SL over 6’SL, whereas SialA appeared to exclusively catalyze synthesis of 3’SL. However, concentrations of these trans-sialylation products never exceeded 0.16 mM (Fig 4), and they were completely hydrolyzed after 30 h. The finding that SialH is the only trans-sialidase among the identified candidates supports the hypothesis that an aromatic sandwich above the active site is a suitable marker for identification of trans-sialidases.

## Conclusions

The rational identification of novel trans-sialidases using a simple computational approach based on a putative acceptor binding sites was explored. The study was based on the hypothesis that an aromatic sandwich present immediately above the active site can be used as a trans-sialidase identification marker. From a database of 2909 sequences coding for genes categorized as sialidases, one candidate–SialH from *H*. *parasuis*–was predicted to have an active site displaying an aromatic sandwich comprised of W366 and F168. Three additional candidates displayed a narrow binding cleft (but no aromatic sandwich) and were included in experimental evaluation of trans-sialidase activity. The results supported the initial hypothesis regarding the importance of the aromatic sandwich with SialH being the only trans-sialidase among the four analyzed candidates. SialH was shown to exhibit a product-to-hydrolysis ratio (R_P,H_) of 2.5, categorizing the enzyme as a trans-sialidase since R_P,H_>1. Surprisingly, a total of three sialylation products were identified during this study, including the novel trans-sialylation product 3SL, which was produced efficiently by SialH (R_3SL,H_ = 0.7). It is the first time that a sequence analysis approach has been successful in identifying a trans-sialidase and the first time a rational approach has led to the discovery of a trans-sialidase outside the *Trypanosoma* genus.

## Supporting Information

S1 Fig Homology modeling of active sites of trans-sialidase candidates falling for criterion 1. Active site and aromatic sandwich of TcTS (gray) aligned to homology models of (putative) sialidases from (a) *Actinomyces* sp. oral taxon 849 str. F0330, (b) *Actinomyces odontolyticus*, (c) *Congregibacter litoralis*, (d) *Diplosphaera colitermitum*, (e) *Oceanobacter* sp., (f) *Opitutaceae bacterium* TAV1, (g) *Opitutaceae bacterium* TAV5, (h) *Pirellula staleyi*, (i) *Verrucomicrobium spinosum*, (j) *Pseudomonas mendocina*, (k) *Ruminococcus albus*, and (l) *Streptomyces griseoflavus*. None of these produced active sites with all nine sialyl interacting residues, thus falling for selection criterion 1.
(PDF)

S2 Fig HPAEC-PAD chromatogram of SialH trans-sialylation products (zoom). The reaction mixture obtained after 90 min of reaction by SialH at 30°C using casein glycomacropeptide (CGMP) as sialyl donor and lactose as acceptor was analysed by HPAEC-PAD and showed and unidentified peak at 9.2 min (blue). External standards of sialic acid (SA; 5.1 min), 6’-sialyllactose (6’SL; 7.7 min), and 3’-sialyllactose (3’SL; 8.2 min) were used (gray). Suspecting that the novel reaction compound was 3-sialyllactose (3SL), the reaction mixture was treated with β-galactosidase, which did indeed cause considerable degradation of the uidentified compound (pink).
(PDF)

S3 Fig ^1^H NMR spectrum (D_2_O, 600 MHz) of SialH trans-sialylation products. To confirm the obtained reaction products, mixed samples were analysed by NMR. As expected, the ^1^H NMR spectrum of the reaction products reveals the presence of 3’-sialyllactose (3’SL) and 6’-sialyllactose (6’SL). Of special interest was the signal at δ 4.537 (Gal H-1) ppm, rendered by the unknown reaction product. Additionally, a possible downfield shift of Glc H-3 (δ 3.56 → δ 3.73 ppm) suggested sialylation at O-3 of the Glc residue. Since there are no extra Neu5Ac axial or equatorial H-3 signal in the ^1^H NMR spectrum, the Neu5Ac signals at δ 1.795 (Neu5Ac H-3a) and δ 2.734 (Neu5Ac H-3e) ppm also suggest an (α2,3)-linked Neu5Ac residue. Thus, the unknown sialyllation product was identified as 3-sialyllactose (3SL).
(PDF)

S1 Table Sequence IDs of the sequences identified in the initial database screening. 46 sequences were identified using the ΩxRDR search motif (Box 1). Of these, 30 were rejected before homology modeling due to redundancy or the fact that they were already known *Trypanosoma* trans-sialidases (TS), including TcTS. Redundancy indicates that there was more than one sequence from the same organism (sequence identities were ≥ 77% and is given in parentheses), and one from each was selected for homology modeling. The remaining 16 sequences were subjected to homology modeling (S2 Table).
(PDF)

S2 Table Sequence IDs of the 3D alignments of the 15 sequences selected for homology modeling (HM). Homology models were aligned to the TcTS crystal structure (PDB 1S0I) for evaluation against selection criteria 1 and 2 (Fig 2; S1 Fig). The reason for rejecting a sequence after homology modeling is indicated. Falling for criterion 1: Does not contain all nine conserved active site residues (S1 Fig). No template: No template available for homology modeling with HHPred. Candidate name in bold (SialA, SialP) indicates that these are sequences fulfilling the two selection criteria and selected for expression. Sequences of SialM and SialH were added after BLAST of sequences of SialA and SialP: of the two closest homologues for each, these two fulfilled both selection criteria.
(PDF)
