# Supplementary material for: It All Starts with a Sandwich: Identification of Sialidases with Trans-Glycosylation Activity
Source: PLoS One. 2016 Jul 1;11(7):e0158434. doi: 10.1371/journal.pone.0158434 (PMC4930215; doi:10.1371/journal.pone.0158434)
Supplement: S1 Fig — Active site and aromatic sandwich of TcTS (gray) aligned to homology models of (putative) sialidases from (a) Actinomyces sp. oral taxon 849 str. F0330, (b) Actinomyces odontolyticus, (c) Congregibacter litoralis, (d) Diplosphaera colitermitum, (e) Oceanobacter sp., (f) Opitutaceae bacterium TAV1, (g) Opitutaceae bacterium TAV5, (h) Pirellula staleyi, (i) Verrucomicrobium spinosum, (j) Pseudomonas mendocina, (k) Ruminococcus albus, and (l) Streptomyces griseoflavus. None of these produced active sites with all nine sialyl interacting residues, thus falling for selection criterion 1. (PDF) [file pone.0158434.s001.pdf]

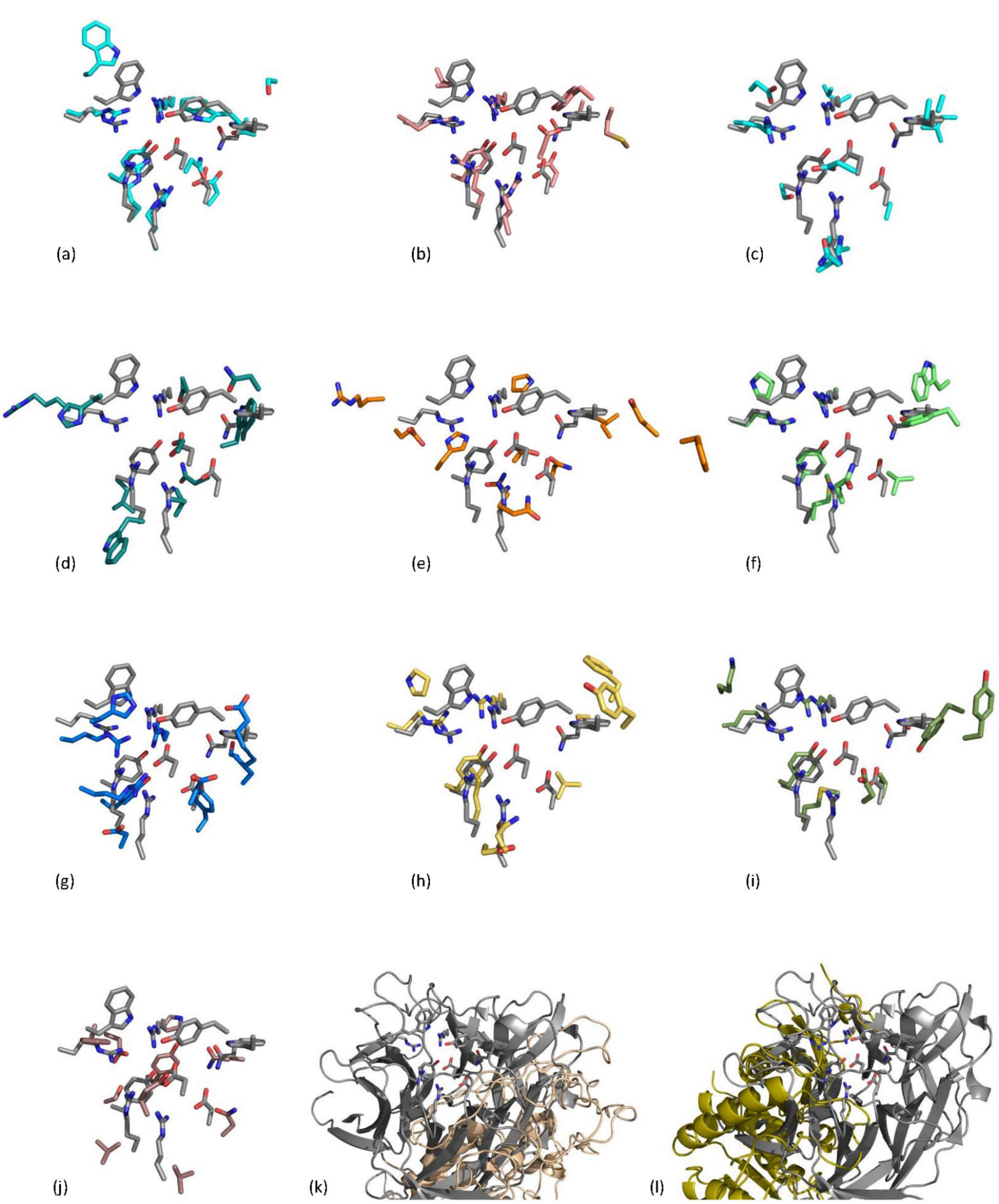

S1 Fig. Homology modeling of active sites of trans-sialidase candidates falling for criterion 1. Active site and aromatic sandwich of TcTS (grey) aligned to homology models of (putative) sialidases from (a) *Actinomyces* sp. oral taxon 849 str. F0330, (b) *Actinomyces odontolyticus*, (c) *Congregibacter litoralis*, (d) *Diplosphaera colitermitum*, (e) *Oceanobacter* sp., (f) *Opitutaceae bacterium* TAV1, (g) *Opitutaceae bacterium* TAV5, (h) *Pirellula staleyi*, (i) *Verrucomicrobium spinosum*, (j) *Pseudomonas mendocina*, (k) *Ruminococcus albus*, and (l) *Streptomyces griseoflavus*. None of these produced active sites with all nine sialyl interacting residues, thus falling for selection criterion 1.
