# Supplementary material for: It All Starts with a Sandwich: Identification of Sialidases with Trans-Glycosylation Activity
Source: PLoS One. 2016 Jul 1;11(7):e0158434. doi: 10.1371/journal.pone.0158434 (PMC4930215; doi:10.1371/journal.pone.0158434)
Supplement: S2 Fig — The reaction mixture obtained after 90 min of reaction by SialH at 30°C using casein glycomacropeptide (CGMP) as sialyl donor and lactose as acceptor was analysed by HPAEC-PAD and showed and unidentified peak at 9.2 min (blue). External standards of sialic acid (SA; 5.1 min), 6’-sialyllactose (6’SL; 7.7 min), and 3’-sialyllactose (3’SL; 8.2 min) were used (gray). Suspecting that the novel reaction compound was 3-sialyllactose (3SL), the reaction mixture was treated with β-galactosidase, which did indeed cause considerable degradation of the uidentified compound (pink). (PDF) [file pone.0158434.s002.pdf]

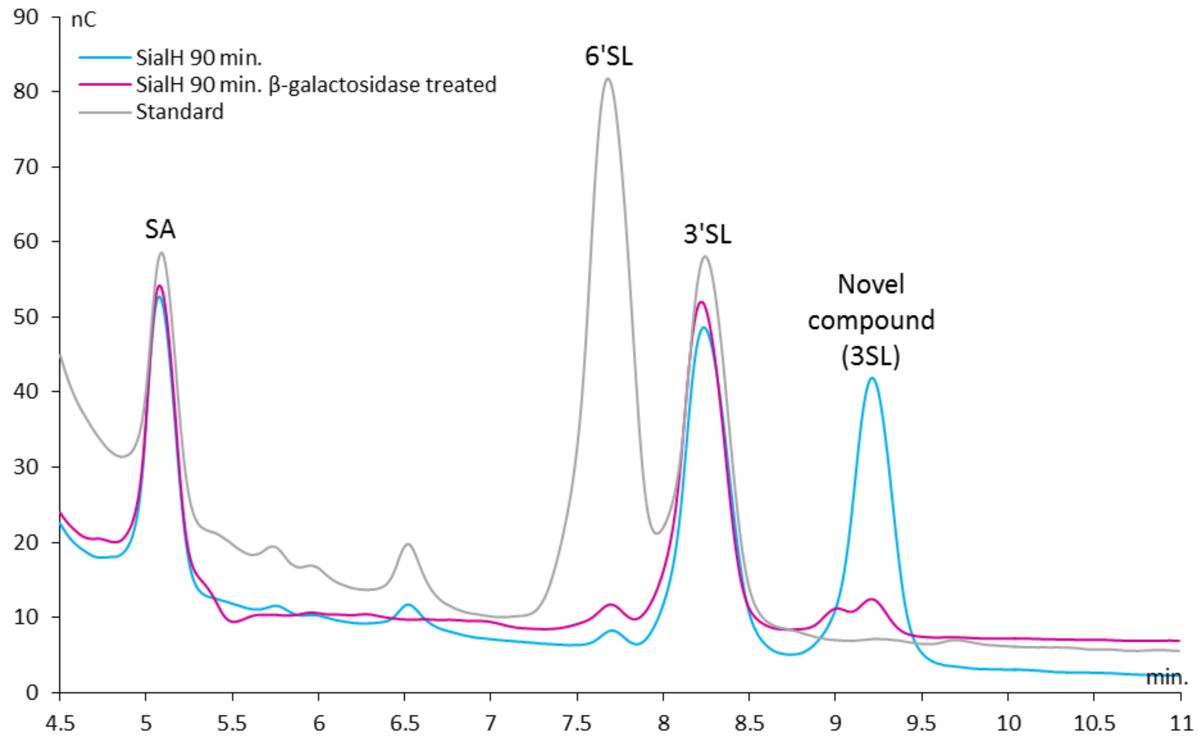

S2 Fig. HPAEC-PAD chromatogram of SialH trans-sialylation products (zoom). The reaction mixture obtained after 90 min of reaction by SialH at 30°C using casein glycomacropeptide (CGMP) as sialyl donor and lactose as acceptor was analysed by HPAEC-PAD and showed and unidentified peak at 9.2 min (blue). External standards of sialic acid (SA; 5.1 min), 6'-sialyllactose (6'SL; 7.7 min), and 3'-sialyllactose (3'SL; 8.2 min) were used (grey). Suspecting that the novel reaction compound was 3-sialyllactose (3SL), the reaction mixture was treated with β-galactosidase, which did indeed cause considerable degradation of the uidentified compound (pink).
