# Supplementary material for: It All Starts with a Sandwich: Identification of Sialidases with Trans-Glycosylation Activity
Source: PLoS One. 2016 Jul 1;11(7):e0158434. doi: 10.1371/journal.pone.0158434 (PMC4930215; doi:10.1371/journal.pone.0158434)
Supplement: S3 Fig — To confirm the obtained reaction products, mixed samples were analysed by NMR. As expected, the 1H NMR spectrum of the reaction products reveals the presence of 3’-sialyllactose (3’SL) and 6’-sialyllactose (6’SL). Of special interest was the signal at δ 4.537 (Gal H-1) ppm, rendered by the unknown reaction product. Additionally, a possible downfield shift of Glc H-3 (δ 3.56 → δ 3.73 ppm) suggested sialylation at O-3 of the Glc residue. Since there are no extra Neu5Ac axial or equatorial H-3 signal in the 1H NMR spectrum, the Neu5Ac signals at δ 1.795 (Neu5Ac H-3a) and δ 2.734 (Neu5Ac H-3e) ppm also suggest an (α2,3)-linked Neu5Ac residue. Thus, the unknown sialyllation product was identified as 3-sialyllactose (3SL). (PDF) [file pone.0158434.s003.pdf]

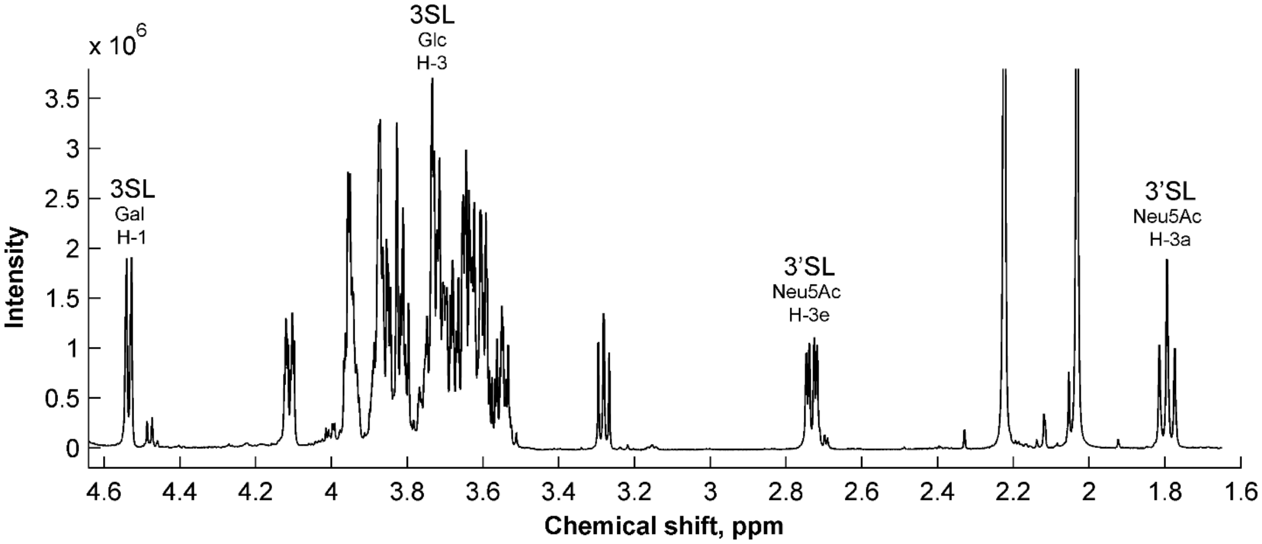

S3 Fig. ^{1}H NMR spectrum (D_2O, 600 MHz) of SialH trans-sialylation products. To confirm the obtained reaction products, mixed samples were analysed by NMR. As expected, the ^{1}H NMR spectrum of the reaction products reveals the presence of 3'-sialyllactose (3'SL) and 6'-sialyllactose (6'SL). Of special interest was the signal at δ 4.537 (Gal H-1) ppm, rendered by the unknown reaction product. Additionally, a possible downfield shift of Glc H-3 (δ 3.56 → δ 3.73 ppm) suggested sialylation at O-3 of the Glc residue. Since there are no extra Neu5Ac axial or equatorial H-3 signal in the ^{1}H NMR spectrum, the Neu5Ac signals at δ 1.795 (Neu5Ac H-3a) and δ 2.734 (Neu5Ac H-3e) ppm also suggest an (α2,3)-linked Neu5Ac residue. Thus, the unknown sialyllation product was identified as 3-sialyllactose (3SL).
