# Supplementary material for: It All Starts with a Sandwich: Identification of Sialidases with Trans-Glycosylation Activity
Source: PLoS One. 2016 Jul 1;11(7):e0158434. doi: 10.1371/journal.pone.0158434 (PMC4930215; doi:10.1371/journal.pone.0158434)
Supplement: S1 Table — 46 sequences were identified using the ΩxRDR search motif (Box 1). Of these, 30 were rejected before homology modeling due to redundancy or the fact that they were already known Trypanosoma trans-sialidases (TS), including TcTS. Redundancy indicates that there was more than one sequence from the same organism (sequence identities were ≥ 77% and is given in parentheses), and one from each was selected for homology modeling. The remaining 16 sequences were subjected to homology modeling (S2 Table). (PDF) [file pone.0158434.s004.pdf]

S1 Table. Sequence IDs of the sequences identified in the initial database screening. 46 sequences were identified using the ΩxRDR search motif. Of these, 30 were rejected before homology modeling due to redundancy or the fact that they were already known *Trypanosoma* trans-sialidases (TS), including TcTS. Redundancy indicates that there was more than one sequence from the same organism (sequence identities were ≥ 77% and is given in parentheses), and one from each was selected for homology modeling. The remaining 16 sequences were subjected to homology modeling (S2 Table).

Sequence	Reason for rejection
>gi|154508657|ref|ZP_02044299.1| hypothetical protein ACTODO_01161 [Actinomyces odontolyticus ATCC 17982]	Redundancy (93%)
>gi|293191770|ref|ZP_06609294.1| putative sialidase [Actinomyces odontolyticus F0309]	
>gi|325067903|ref|ZP_08126576.1| BNR/Asp-box repeat protein [Actinomyces oris K20]	
>gi|329944094|ref|ZP_08292353.1| BNR/Asp-box repeat protein [Actinomyces sp. oral taxon 170 str. F0386]	Redundancy (85%)
>gi|329945272|ref|ZP_08293083.1| hypothetical protein HMPREF9056_00968 [Actinomyces sp. oral taxon 170 str. F0386]	
>gi|320531595|ref|ZP_08032538.1| BNR/Asp-box repeat protein [Actinomyces sp. oral taxon 171 str. F0337]	Redundancy (84%)
>gi|343521845|ref|ZP_08758811.1| BNR/Asp-box repeat protein [Actinomyces sp. oral taxon 175 str. F0384]	Redundancy (77%)
>gi|365828654|ref|ZP_09370449.1| hypothetical protein HMPREF0975_02232 [Actinomyces sp. oral taxon 849 str. F0330]	
>gi|326773842|ref|ZP_08233124.1| sialidase (Neuraminidase) [Actinomyces viscosus C505]	Redundancy (97%)*
>gi|256379736|ref|YP_003103396.1| hypothetical protein Amir_5736 [Actinosynnema mirum DSM 43827]	
>gi|94501941|ref|ZP_01308450.1| BNR/Asp-box repeat protein [Oceanobacter sp. RED65]	
>gi|88703428|ref|ZP_01101144.1| sialidase family protein [Congregibacter litoralis KT71]	
>gi|88703428|ref|ZP_01101144.1| sialidase family protein [Congregibacter litoralis KT71]	Redundancy (100%)
>gi|88703428|ref|ZP_01101144.1| sialidase family protein [Congregibacter litoralis KT71]	Redundancy (100%)
>gi|88703428|ref|ZP_01101144.1| sialidase family protein [Congregibacter litoralis KT71]	Redundancy (100%)
>gi|88703428|ref|ZP_01101144.1| sialidase family protein [Congregibacter litoralis KT71]	Redundancy (100%)
>gi|225156471|ref|ZP_03724806.1| hypothetical protein ObacDRAFT_8294 [Diplosphaera colitermitum TAV2]	
>gi|390121970|ref|ZP_10215489.1| hypothetical protein ObacTA_17328 [Opitutaceae bacterium TAV1]	
>gi|373852611|ref|ZP_09595411.1| hypothetical protein Opit5DRAFT_3465 [Opitutaceae bacterium TAV5]	
>gi|378774370|ref|YP_005176613.1| sialidase [Pasteurella multocida 36950]	Redundancy (99%)
>gi|386834192|ref|YP_006239507.1| sialidase NanH [Pasteurella multocida subsp. multocida str. 3480]	
>gi|283782130|ref|YP_003372885.1| hypothetical protein Psta_4378 [Pirellula staleyi DSM 6068]	
>gi|330501037|ref|YP_004377906.1| BNR/Asp-box repeat-containing protein [Pseudomonas mendocina NK-01]	
>gi|319788706|ref|YP_004090021.1| Dockerin type 1 [Ruminococcus albus 7]	
>gi|302557686|ref|ZP_07310028.1| conserved hypothetical protein [Streptomyces griseoflavus Tu4000]	

>gi|72388886|ref|XP_844738.1| trans-sialidase [Trypanosoma brucei brucei strain 927/4 GUTat10.1]	Known TS
>gi|72392022|ref|XP_846305.1| trans-sialidase [Trypanosoma brucei brucei strain 927/4 GUTat10.1]	Known TS
>gi|72393525|ref|XP_847563.1| trans-sialidase [Trypanosoma brucei brucei strain 927/4 GUTat10.1]	Known TS
>gi|72393527|ref|XP_847564.1| trans-sialidase [Trypanosoma brucei brucei strain 927/4 GUTat10.1]	Known TS
>gi|71403511|ref|XP_804548.1| trans-sialidase [Trypanosoma cruzi strain CL Brener]	TcTS
>gi|71407532|ref|XP_806228.1| trans-sialidase [Trypanosoma cruzi strain CL Brener]	TcTS
>gi|71408056|ref|XP_806454.1| trans-sialidase [Trypanosoma cruzi strain CL Brener]	TcTS
>gi|71409458|ref|XP_807074.1| trans-sialidase [Trypanosoma cruzi strain CL Brener]	TcTS
>gi|71409982|ref|XP_807308.1| trans-sialidase [Trypanosoma cruzi strain CL Brener]	TcTS
>gi|71410253|ref|XP_807431.1| trans-sialidase [Trypanosoma cruzi strain CL Brener]	TcTS
>gi|71410408|ref|XP_807499.1| trans-sialidase [Trypanosoma cruzi strain CL Brener]	TcTS
>gi|71410614|ref|XP_807593.1| trans-sialidase [Trypanosoma cruzi strain CL Brener]	TcTS
>gi|71412760|ref|XP_808550.1| trans-sialidase [Trypanosoma cruzi strain CL Brener]	TcTS
>gi|71422272|ref|XP_812082.1| trans-sialidase [Trypanosoma cruzi strain CL Brener]	TcTS
>gi|71423719|ref|XP_812546.1| trans-sialidase [Trypanosoma cruzi strain CL Brener]	TcTS
>gi|71649806|ref|XP_813615.1| trans-sialidase [Trypanosoma cruzi strain CL Brener]	TcTS
>gi|71649937|ref|XP_813677.1| trans-sialidase [Trypanosoma cruzi strain CL Brener]	TcTS
>gi|71653254|ref|XP_815267.1| trans-sialidase [Trypanosoma cruzi strain CL Brener]	TcTS
>gi|71655936|ref|XP_816523.1| trans-sialidase [Trypanosoma cruzi strain CL Brener]	TcTS
>gi|71660101|ref|XP_821769.1| trans-sialidase [Trypanosoma cruzi strain CL Brener]	TcTS
>gi|171913862|ref|ZP_02929332.1| hypothetical protein VspiD_21825 [Verrucomicrobium spinosum DSM 4136]	

*This sequence was 97% identical to the one from *Actinomyces oris*, which was finally selected for expression after fulfilling both criteria (SialA). The one from *A. oris* was selected because of its smaller size (the main difference between the two is the C-terminal of 226 amino acids found in the sialidase from *A. viscosus*).
