# Supplementary material for: It All Starts with a Sandwich: Identification of Sialidases with Trans-Glycosylation Activity
Source: PLoS One. 2016 Jul 1;11(7):e0158434. doi: 10.1371/journal.pone.0158434 (PMC4930215; doi:10.1371/journal.pone.0158434)
Supplement: S2 Table — Homology models were aligned to the TcTS crystal structure (PDB 1S0I) for evaluation against selection criteria 1 and 2 (Fig 2; S1 Fig). The reason for rejecting a sequence after homology modeling is indicated. Falling for criterion 1: Does not contain all nine conserved active site residues (S1 Fig). No template: No template available for homology modeling with HHPred. Candidate name in bold (SialA, SialP) indicates that these are sequences fulfilling the two selection criteria and selected for expression. Sequences of SialM and SialH were added after BLAST of sequences of SialA and SialP: of the two closest homologues for each, these two fulfilled both selection criteria. (PDF) [file pone.0158434.s005.pdf]

**S2 Table. Sequence IDs of the 3D alignments of the 15 sequences selected for homology modeling (HM).** Homology models were aligned to the TcTS crystal structure (PDB 1S0I) for evaluation against selection criteria 1 and 2 (Fig 2; S1 Fig). The reason for rejecting a sequence after homology modeling is indicated. Falling for criterion 1: Does not contain all nine conserved active site residues (S1 Fig). No template: No template available for homology modeling with HHPred. Candidate name in bold (SialA, SialP) indicates that these are sequences fulfilling the two selection criteria and selected for expression. Sequences of SialM and SialH were added after BLAST of sequences of SialA and SialP: of the two closest homologues for each, these two fulfilled both selection criteria.

| Sequence | Reason for rejection after HM |
|---|---|
| >gi\|293191770\|ref\|ZP_06609294.1\| putative sialidase [Actinomyces odontolyticus F0309] | Criterion 1 |
| >gi\|325067903\|ref\|ZP_08126576.1\| BNR/Asp-box repeat protein [Actinomyces oris K20] | **SialA** |
| >gi\|365828654\|ref\|ZP_09370449.1\| hypothetical protein HMPREF0975_02232 [Actinomyces sp. oral taxon 849 str. F0330] | Criterion 1 |
| >gi\|329945272\|ref\|ZP_08293083.1\| hypothetical protein HMPREF9056_00968 [Actinomyces sp. oral taxon 170 str. F0386] | No template |
| >gi\|256379736\|ref\|YP_003103396.1\| hypothetical protein Amir_5736 [Actinosynnema mirum DSM 43827] | No template |
| >gi\|94501941\|ref\|ZP_01308450.1\| BNR/Asp-box repeat protein [Oceanobacter sp. RED65] | Criterion 1 |
| >gi\|88703428\|ref\|ZP_01101144.1\| sialidase family protein [Congregibacter litoralis KT71] | Criterion 1 |
| >gi\|225156471\|ref\|ZP_03724806.1\| hypothetical protein ObacDRAFT_8294 [Diplosphaera colitermitum TAV2] | Criterion 1 |
| >gi\|390121970\|ref\|ZP_10215489.1\| hypothetical protein ObacTA_17328 [Opitutaceae bacterium TAV1] | Criterion 1 |
| >gi\|373852611\|ref\|ZP_09595411.1\| hypothetical protein Opit5DRAFT_3465 [Opitutaceae bacterium TAV5] | Criterion 1 |
| >gi\|386834192\|ref\|YP_006239507.1\| sialidase NanH [Pasteurella multocida subsp. multocida str. 3480] | **SialP** |
| >gi\|283782130\|ref\|YP_003372885.1\| hypothetical protein Psta_4378 [Pirellula staleyi DSM 6068] | Criterion 1 |
| >gi\|330501037\|ref\|YP_004377906.1\| BNR/Asp-box repeat-containing protein [Pseudomonas mendocina NK-01] | Criterion 1 |
| >gi\|319788706\|ref\|YP_004090021.1\| Dockerin type 1 [Ruminococcus albus 7] | Criterion 1 |
| >gi\|302557686\|ref\|ZP_07310028.1\| conserved hypothetical protein [Streptomyces griseoflavus Tu4000] | Criterion 1 |
| >gi\|171913862\|ref\|ZP_02929332.1\| hypothetical protein VspiD_21825 [Verrucomicrobium spinosum DSM 4136] | Criterion 1 |

**Added after BLAST of SialA and SialP**

| | |
|---|---|
| gi\|261493088\|ref\|ZP_05989626.1\| neuraminidase [Mannheimia haemolytica serotype A2 str. BOVINE] | **SialM** |
| gi\|167854931\|ref\|ZP_02477707.1\| hypothetical protein HPS_05673 [Haemophilus parasuis 29755] | **SialH** |
